# Real-time tracking of three-dimensional atomic dynamics of Pt trimer on TiO_2_ (110)

**DOI:** 10.1126/sciadv.adk6501

**Published:** 2024-02-28

**Authors:** Ryo Ishikawa, Toshihiro Futazuka, Yu Jimbo, Kazuaki Kawahara, Naoya Shibata, Yuichi Ikuhara

**Affiliations:** ^1^ Institute of Engineering Innovation, University of Tokyo, Bunkyo, Tokyo 113-8656, Japan.; ^2^ EM Research and Development, JEOL Ltd., Akishima, Japan.; ^3^ Nanostructures Research Laboratory, Japan Fine Ceramics Center, Nagoya, Aichi 456-8587, Japan.

## Abstract

Single and multi-atoms supported on oxide substrates ultimately increase the efficiency of noble metal atom use, and moreover, catalytic activity and selectivity are also improved substantially. However, single and multi-atoms are unstable under catalytic conditions, and these metal atoms spontaneously aggregate and grow into nanoparticles. Catalytic performance is strongly related to local atomic configurations, and hence, it is essential to determine the three-dimensional (3D) atomic structures of multi-atoms on the substrate and their structural dynamics. Here, we show the real-time tracking of the 3D structural evolution of a Pt trimer on TiO_2_ (110) substrate at a high temperature, using high-spatiotemporal-resolution scanning transmission electron microscopy, where sub-angstrom spatial resolution is maintained, while the temporal resolution reaches 40 milliseconds. With the aid of prior structural knowledge of a Pt trimer for 3D reconstruction, the present method could open the way to characterize in situ atomic-scale structural dynamics, especially meta-stable structural transition.

## Teaser

3D structural evolution of a Pt trimer on TiO_2_ (110) is tracked in real time by high-spatiotemporal-resolution STEM.

## INTRODUCTION

When downsizing noble metal nanoparticles (NPs) supported on oxide substrates, the catalytic activity and selectivity per metal atom increases, and hence, single or multi-atom catalysis can potentially maximize the efficiency of various chemical reactions (*1*–*3*). However, single-metal atoms are usually unstable during the chemical reaction and spontaneously aggregate or grow into dimers, trimers, and subnanoclusters, assisted by their high mobility on the substrate surface. Although several excellent synthesis methods have been proposed, such as anchoring single-metal atoms onto metal oxides (*1*, *4*), the single-metal atoms in most catalytic systems still aggregate under a reduced atmosphere and high temperatures, leading to substantial degradation of catalytic activity (*2*). In addition to the size effects, the catalytic activity and selectivity are strongly related to the local atomic coordination of the catalytic metal atoms, such as coordination numbers and interatomic distances (*5*). It is therefore essential to determine three-dimensional (3D) atomic environments and trace the dynamics of single or multi-metal atoms on oxide surfaces, which remains a major challenge.

Atomic-resolution annular dark-field scanning transmission electron microscopy (ADF STEM) has been used to determine local atomic structures with sub-angstrom spatial resolution, and it has a unique capability to visualize even single atoms directly (*6*, *7*). However, electron microscopy has two major difficulties for real-time tracking of the 3D structural dynamics of multi-atoms. First, owing to the scanning nature of the electron probe, the temporal resolution in atomic-resolution STEM has been limited to a few seconds in a standard field of view (512 × 512 pixels) (*8*, *9*). Second, the atomic structure information from a single STEM image is limited to the projected two dimensions. Although it is possible to reconstruct 3D atomic structures via electron tomography (*10*) and depth sectioning (*11*), it requires multiple atomic-resolution STEM images. Thus, the temporal resolution will be a few minutes to hours. To resolve the first issue, we have recently developed a new high-speed scanning probe system with a pair of extremely low-inductance electromagnetic scan coils combined with a short lifetime scintillator (*12*), and temporal resolution in atomic-resolution STEM reaches 40 ms, corresponding to 25 frames per second (fps). To overcome the second issue, we develop an algorithm to reconstruct the 3D atomic structure of Pt multi-atoms from a single STEM image by solving nonlinear equations under a reasonable constraint. Here, we show the real-time tracking of 3D atomic structural dynamics of a Pt trimer supported on the TiO_2_ (110) using high-spatiotemporal-resolution STEM (HSTR-STEM). The respective 3D atomic structures of the Pt trimer are reconstructed from single ADF STEM images at a temporal resolution of 40 ms. The validity of the reconstructed 3D atomic structures and their energetic stability are evaluated by density functional theory (DFT) calculations. We elucidate that the structural dynamics of the Pt trimer on the TiO_2_ (110) can be described by 3D rotations (Euler angles) and center-of-mass (CoM) translations because the interatomic distance of the Pt trimer is almost constant. Under the condition, the Pt trimer undergoes energetically meta-stable structures as a transition state. The lifetime of the transition-state structure is considerably short, and hence, HSTR-STEM imaging should be essential to directly observe the real-time structural dynamics.

## RESULTS

### Tracking of a Pt trimer on TiO_2_ (110) surface

**Fig. 1.**
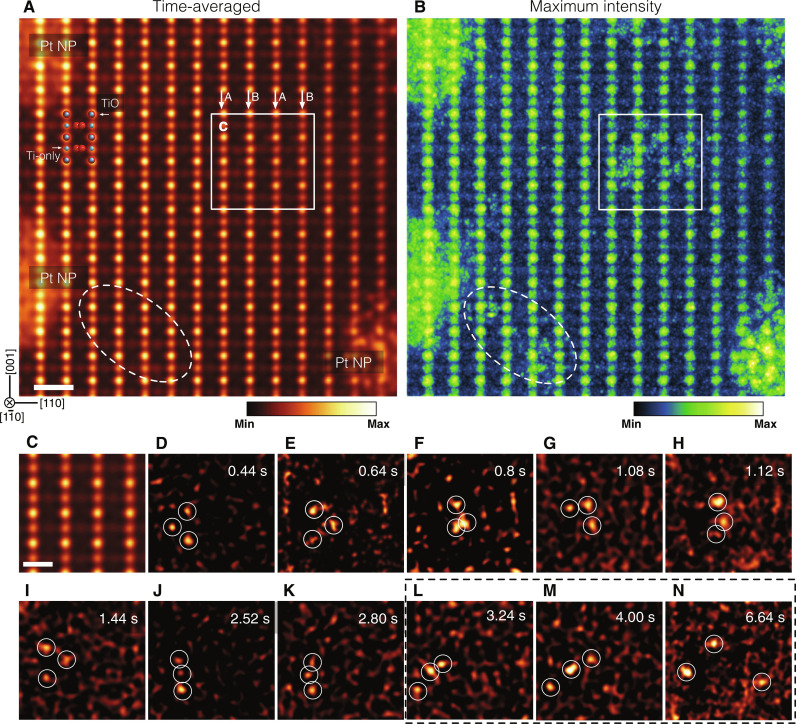
Real-time tracking of the Pt trimer on the TiO_2_ (110) surface. (**A**) Time-averaged (672 frames) atomic-resolution ADF STEM image viewed along the TiO_2_ [1-10] direction. Three bright islands correspond to the Pt NPs. (**B**) Maximum intensity map constructed from the whole time-sequential frames. (**C**) Enlarged image of the white rectangle in (A). (**D** to **N**) The locations of three Pt atoms indicated by white circles, where the contrast from the TiO_2_ substrate is removed by subtracting the image of (C) from respective frames. The time stamps are given on the right top for the respective panels. Scale bars, 5 Å (A) and 3 Å (C).

Pt atoms were dispersed on an atomically flat rutile TiO_2_ (110) surface in an ultrahigh-vacuum chamber (*13*, *14*). To amplify the visibility of Pt single atoms on the TiO_2_ (110) in ADF STEM imaging, we used the large illumination semiangle of 40 mrad at 300 kV that has a higher depth resolution (*11*). To track the dynamics of Pt atoms on the TiO_2_ (110), we time-sequentially acquired 672 frames in 26.9 s, where the sample was heated at 573 K in the electron microscope to promote the motion of Pt atoms (see movie S1 compressed data). Figure 1A shows the full-time–averaged ADF STEM image viewed along the TiO_2_ [1-10] direction. Owing to the *Z*-contrast nature (*Z* is the atomic number) of the ADF STEM image, the most and second brightest dots correspond to TiO and Ti-only atomic columns, respectively, as shown by the overlaid structure model. Three bright islands at the left and right edges in Fig. 1A indicate Pt NPs. At 573 K, these Pt NPs are not well crystallized, and the atomic structures of these Pt NPs are dynamically changed during the observations, and hence, we cannot see clear atomic structures of Pt NPs in the time-averaged image. For the same reason, the moving Pt single atomic contrast is washed out, and we cannot see the single Pt atoms in the time-averaged image.

Because the observation was performed at a considerably low dose condition (2624 e^−^ Å^−2^) due to the extremely short acquisition time of 83 ns/pixel, the signal-to-noise ratio of a single frame is considerably low, and thereby, it becomes a major challenge to track Pt atoms. We first constructed the maximum intensity map (Fig. 1B) to find the locations of the Pt atoms, where the maximum intensity from the whole frames was selected for the respective pixels (see Materials and Methods). As indicated by a white rectangle and ellipse in Fig. 1B, plenty of brighter dots were observed. Hereafter, we focus on the white rectangle region containing a Pt trimer. To enhance the visibility of single Pt atoms, we remove the intensity of the TiO_2_ substrate from respective frames by subtracting the time-averaged image of Fig. 1C (see movie S2), and representative frames are shown in Fig. 1 (D to K). We note that, during the observation, the sample was heated at 573 K, which introduces nonlinear local specimen drift. This imperfection of local drift correction and quantum noise due to the low electron dose gives residual background noise in the subtracted frames of Fig. 1 (D to K). Therefore, *Z*-contrast intensities at some Pt atoms (e.g., Fig. 1H) become similar to the background noise. To rigidly determine such Pt atom locations, we doubly checked the Pt atom locations in both the subtracted and the denoised original frames. Furthermore, we also confirmed the validity of the Pt atom locations by comparing with before and after frames. With these criteria, we determine the Pt atom locations, and three Pt atoms are overlaid by the white circles, and the projected locations of single Pt atoms are precisely determined by 2D Gaussian fitting. The structure of the Pt trimer rapidly varies as a function of time, but three Pt atoms are well correlated during the dynamic structural transition. Although several single Pt atoms were emitted from the Pt NPs, the Pt trimer was well isolated from the other single Pt atoms. This is most evident from the maximum intensity map of Fig. 1B, where the white rectangle region is not connected to the other bright contrasts of single Pt atoms. After 3.1 s (Fig. 1, L to N), the distances between Pt atoms suddenly increase, and these Pt atoms may behave as isolated atoms. However, it is still unclear whether three Pt atoms are bonded or not bonded because it is difficult to measure the 3D interatomic distance from the projected 2D images. To overcome this issue, it is essential to obtain the 3D atomic configuration of the Pt trimer from the 2D projection images.

### 3D reconstruction of a Pt trimer

**Fig. 2.**
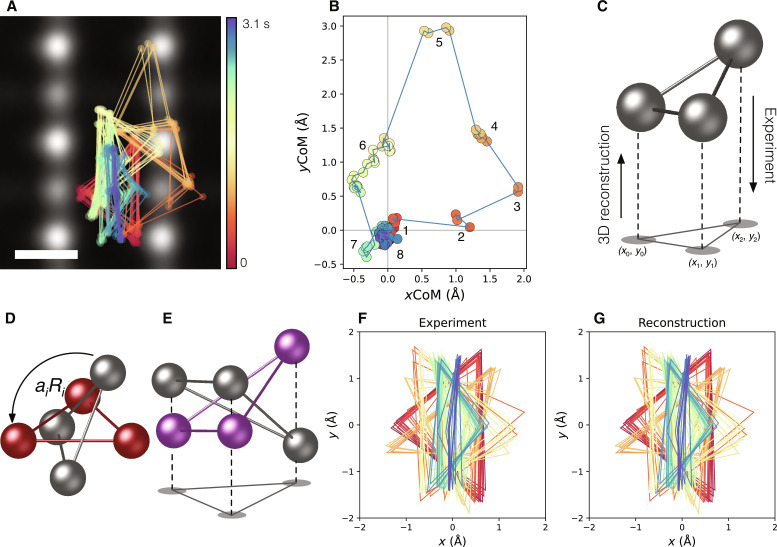
3D reconstruction of the Pt trimer on the TiO_2_ (110) from 2D projected atomic-resolution images. (**A**) Traces of the Pt trimer projected onto TiO_2_ (110) as a function of time. (**B**) CoM translation of the Pt timer as a function of time, where the colors correspond to time as shown in (A). (**C** to **E**) Schematic views of 3D reconstruction of the Pt trimer from the 2D projected image. (**F** and **G**) Experimental traces of the Pt trimer and 2D projected traces from the 3D reconstructed Pt trimer as a function of time, respectively, where respective CoM is set to the origin. Scale bar, 2 Å (A).

Figure 2A shows the 78 traces of the Pt trimer projected onto TiO_2_ (110) plane (*xy* plane) as a function of time (0.00 to 3.08 s), which are overlaid on the time-averaged ADF STEM image. Figure 2B shows the in-plane translations of the Pt trimer CoM as a function of time: *x*- and *y*-translation ranges are −0.5 Å < *x* < 1.9 Å and −3.0 Å < *y* < 0.4 Å, respectively (*x* and *y* correspond to [110] and [001] directions). Although the observed CoM translation range of the Pt trimer is relatively narrow, the area covers two unit cells of TiO_2_. It is therefore enough to comprehensively explore the stable atomic configurations and the dynamics of the Pt trimer on the TiO_2_ (110) surface. As indicated by the numbers in Fig. 2B, the movements of the Pt trimer on the TiO_2_ (110) surface are not continuous but discrete, suggesting several energetically favorable atomic configurations. The structures can be classified into eight types, which are given in Fig. 1 (D to K).

According to the previous DFT calculations, it is predicted that the stable Pt trimer structures on the anatase TiO_2_ (101) surface have equilateral triangles (*15*). Using the constraint of an equilateral triangle for the present Pt trimer, the 3D structures could be reconstructed from three pairs of 2D projected Pt atom positions. Later, we will discuss the validity of our assumption by DFT calculations in our system. An equilateral triangle in 3D may be described by the four structure parameters: the interatomic distance and three Euler angles of {*a*, θ, ϕ, ψ}. As shown in Fig. 2C, we can experimentally measure a set of six independent parameters as a function of time: three pairs of projected Pt atom positions *X_i_*, where *i* is the index for the *i*th frame. By applying a suitable stretch (*a*) and a 3D rotation matrix (θ, ϕ, ψ) to a reference equilateral triangle (red triangle in Fig. 2D), the 2D projected components of [*a_i_R_i_*(θ*_i_*, ϕ*_i_*, ψ*_i_*)*X*_0_]_2D_ will be matched with the measured *X_i_* components, where *R_i_*(θ*_i_*, ϕ*_i_*, ψ*_i_*) is a 3D rotation matrix, and *X*_0_ is a set of 3D positions of a reference equilateral triangle (see Materials and Methods). Here, we solve the above four nonlinear equations by minimizing the loss function

$$L=\text{argmi}n_{i}\;\left\{{|X_{i}-{\left[a_{i}R_{i}\left(\theta_{i},\phi_{i},\psi_{i}\right)X_{0}\right]}_{2D}|}^{2}\right\}$$ (1)

where the minimization is achieved by a nonlinear optimization algorithm (Broyden-Fletcher-Goldfarb-Shanno algorithm, see Materials and Methods). Figure 2 (F and G) shows the experimentally measured *X_i_* and the 2D projected triangles of [*a_i_R_i_X*_0_]_2D_ obtained by Eq. 1, where the CoM for each triangle is set to the origin. All the reconstructed 3D triangles are well matched with the experimental *X_i_*, suggesting that our constraint is reasonable in the present Pt trimer structural analysis. However, one cannot distinguish the mirror-symmetric 3D atomic structures of Pt trimers in the *xy* plane (see Fig. 2E), although one can recognize the other mirror-symmetric Pt trimer in the *yz* and *xz* planes. To strictly determine the 3D atomic structures of the Pt trimer, we performed systematic DFT calculations to evaluate the adsorption energies of the Pt trimers on the TiO_2_ (110) surface.

### Structural and energetic evaluation by DFT

**Fig. 3.**
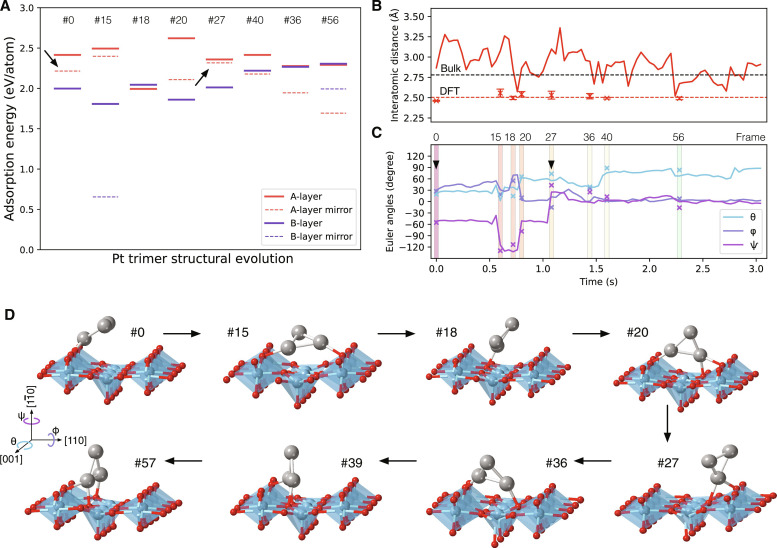
The evolution of structure parameters of 3D Pt trimer and their energetics by DFT calculation. (**A**) Absorption energies per Pt atom (eV/atom) for eight representative Pt structures on the TiO_2_ (110). There are two surface terminations of the TiO_2_ (110) surface: O termination at the A layer (solid red lines) and B layer (solid blue lines), respectively. The dotted lines correspond to *xy*-mirror-symmetric Pt trimer structures, respectively. The higher absorption energy corresponds to higher structural stability. (**B** and **C**) 3D reconstructed structure parameter: interatomic distance in (A) and Euler angles in (C). The structure parameters estimated by DFT calculations are given by cross marks. (**D**) Structural dynamics of the Pt trimer on the TiO_2_ (110) for respective frames.

In addition to *xy*-mirror-symmetric Pt trimer structures, we must consider the surface termination of the TiO_2_ (110) (*16*). According to the truncated surface of TiO_2_ (110), two possible surface terminations are indistinguishable from the projected image of Fig. 1A: O-termination and Ti-termination layers are alternatively arranged along the [110] direction, as indicated by A and B layers in Fig. 1A (see also Fig. 3D). Considering the above four possible atomic configurations, we performed DFT calculations and evaluated the adsorption energies of the Pt trimers onto the TiO_2_ (110) surface, where the experimentally reconstructed 3D atomic structures of the Pt trimer were used as input structure models. Figure 3A shows the adsorption energies per Pt atom (eV/atom) for eight classified structures with four possible configurations: O-termination at the A layer (solid red lines) or the B layer (solid blue lines) and *xy*-mirror symmetric Pt trimers for A or B layers (dashed lines). We note that the higher adsorption energy corresponds to higher structural stability (see Materials and Methods). The solid red lines are basically the most energetically stable for all the configurations, and hence, the surface termination for A and B layers is determined to be O and Ti terminations, respectively.

Using the optimization algorithm of Eq. 1, we determine the 3D interatomic distance and Euler angles of the Pt trimer as a function of time, as shown in Fig. 3 (B and C, respectively). The DFT calculated structure parameters (cross-marks) are also given in Fig. 3 (B and C). The reconstructed interatomic distance of the Pt trimer is experimentally measured to be 2.91 ± 0.17 Å (SEM), which is close to the experimental interatomic distance in the bulk (2.78 Å) at 573 K (*17*), as indicated by a dotted black line. However, the calculated interatomic distance is largely underestimated to be 2.50 ± 0.04 Å (dashed line, at 0 K), although the DFT calculated Pt interatomic distance in the bulk (2.81 Å) is well matched with the experiment (*17*). This discrepancy indicates that the Pt trimer’s coefficient of thermal expansion could be much greater than that in the bulk. The error bars in Fig. 3B correspond to the SEM of DFT calculated three interatomic distances for respective Pt trimers, and the error is negligibly small as ±0.02 Å, suggesting that the Pt trimer must be an equilateral triangle during the movement on the TiO_2_ (110) surface. Therefore, even with the formation of chemical bonding between Pt and Ti/O atoms, the structure of the Pt trimer is rigid, and the 3D atomic dynamics of the Pt trimer can be fully described by the two sets of parameters: CoM translations and Euler angles.

Although we cannot distinguish the *xy*-mirror-symmetric structures in the experimental 3D reconstruction, the DFT structural optimization could largely change the Euler angles of unstable input structures. Therefore, we may uniquely identify the Pt trimer structures by comparing the structure parameters between the experiment and the DFT calculations. As shown in Fig. 3C, the reconstructed Euler angles are relatively well matched with those in the most energetically stable structures (solid red lines in Fig. 3A). For frames #0 and #27 (arrowheads in Fig. 3C), the Euler angles of the most energetically stable structures are largely different from the experiment, where the most energetically stable Euler angles for frames #0 and #27 are (θ, ϕ, ψ) = (8.1, −5.0, −60.5) and (47.8, −39.5, −11.8), respectively. However, the other *xy*-mirror-symmetric structures (dotted red lines in Fig. 3A) are rather matched with experiments. In the present HSTR-STEM imaging, we can trace dynamical structural transition, so it is natural to undergo transition-state (or metal-stable) structures during the dynamic process. We note that the observed mismatches in Fig. 3C should be attributed to underestimating the theoretical interatomic distances. Although there are theoretical reports on the energetic stability of a single Pt atom on the TiO_2_ (110) surface, it will be difficult to predict the stable configurations of the Pt trimer from the extension of the adsorption sites for single Pt atom cases. Because the interatomic distance of the Pt trimer is fixed, one or two Pt atoms of the trimer need to locate at unstable adsorption sites.

## DISCUSSION

For the understanding of the structural dynamics visually and quantitatively, we show the structural evolution of the Pt trimer on the TiO_2_ (110) surface in Fig. 3D. Because the metallic atoms on oxide surfaces can easily lose their outer electrons (*18*), the Pt atoms will act as cations on the oxide surface. The Pt atoms, therefore, prefer to bond with O atoms rather than Ti atoms, which is confirmed for all the structures in Fig. 3D. The average Pt─O bond length is 2.15 ± 0.04 Å, which is close to the Pt─O bond length (2.02 Å) in PtO_2_ (*19*). Although the formation of Pt─O bonding is critical to stabilizing the Pt trimer structure, only one Pt atom can bond with the neighboring O atoms in frames #0, #18, and #27. At these configurations, the remaining two Pt atoms should avoid Pt─Ti bonding, and hence, the Euler angles of θ or ϕ (out of plane) become larger. In frames #15, #20, and #36, two Pt atoms are well-bonded with O atoms, which make the structure energetically stable. However, during the transition process between these stable structures, it becomes essential to go through transition-state structures corresponding to frames #18 and #27, where single Pt atoms are bonded with O atoms. The lifetimes of such structures are usually short, i.e., 80 ms for the structure of frame #18, and we successfully captured the transition-state structure using HSTR-STEM imaging. Until frame #77, the Pt-Pt interatomic distance was maintained at 2.91 ± 0.17 Å. However, the interatomic distance was suddenly increased to greater than 4 Å, and then, these Pt atoms started to move independently on the TiO_2_ (110) surface, which can be seen in Fig. 1 (L to N). Once the Pt─Pt metallic bond is broken in the Pt trimer, the mobility of single Pt atoms becomes much faster than that in the Pt trimer, and we experimentally confirmed that small clustering of Pt atoms could improve the energetic stability.

In summary, we have demonstrated real-time tracking of 3D atomic dynamics of the Pt trimer on the TiO_2_ (110) surface at the temporal resolution of 40 ms, using HSTR-STEM imaging and 3D reconstruction by a nonlinear optimization algorithm combined with DFT calculations. With the present high-spatiotemporal resolution, it becomes possible to track the energetically meta-stable transition-state structures. In this study, we use prior knowledge for 3D reconstruction of Pt trimer (equilateral triangle) to substantially improve a temporal resolution, which may limit universal applications for 3D atomic dynamics, especially for irregular (nonsymmetric) or larger clusters. However, multi-atom clusters could have highly symmetric structures. Therefore, combining with DFT calculations, it could be possible to reconstruct 3D atomic structures of multi-atom clusters from single frames. The present HSTR-STEM imaging will open the way for characterizing real-time atomic structural dynamics, especially atmosphere-controlled in situ experiments.

## MATERIALS AND METHODS

### Preparation of TEM specimen for electron microscopy

A commercially available single crystal of rutile TiO_2_ (110) substrate (SHINKOSHA) was cut into a 3-mm half-disk in diameter. The half disk was mechanically polished and further thinned by the Ar-ion beam milling. The half-disk TEM sample was annealed at 573 K for 1 hour to remove hydrocarbon contamination on the surface and then annealed in the air at 1223 K for 10 hours. With this annealing, we obtained a step-terrace TiO_2_ (110) surface confirmed by topographical imaging in atomic force microscopy. The TEM sample was then transferred into an ultrahigh vacuum chamber (5 × 10^−7^ Pa), and Pt NPs were dispersed onto the step-terrace TiO_2_ (110) surface by a vacuum evaporator (Pascal Co. Ltd.), where the total amount of loaded Pt was 0.6 ml (*14*). Therefore, Pt atoms should be initially loaded onto the TEM sample of the TiO_2_ (110) surface. We then annealed the TEM sample up to 573 K in a microscope. According to our previous report in the same system, we observed Pt NPs on the TiO_2_ (110) surface from the cross-sectional direction (*14*). As increasing the heat temperature of Pt/TiO_2_ (110), we observed the growth of Pt on the TiO_2_ (110) surface, and the Pt atoms are always on the TiO_2_ (110) surface. When the sample was annealed at 973 K for 3 hours, Pt NPs were partly impregnated into the TiO_2_ substrate. However, the present experimental temperature is much lower and short annealing time, and moreover, Pt trimer should be very difficult to correlatively diffuse in the TiO_2_ lattice. Therefore, the observed Pt trimer should be located on the TiO_2_ (110) surface.

### High-spatiotemporal-resolution STEM

After Pt loading, the TEM sample was set in an in situ heating TEM holder (JEOL Ltd.) and loaded into TEM (ARM300CF, JEOL Ltd.). The in situ heating holder was preannealed without the TEM sample in a high vacuum at 1073 K for 2 hours to remove metal-oxide contamination entirely. To thoroughly avoid hydrocarbon contamination and specimen charging, all the TEM observations were performed at 523 K. Atomic-resolution ADF STEM images were acquired by ARM300CF installed at the University of Tokyo, equipped with a cold field-emission gun and a delta-type corrector (*20*). The illumination semiangle is 40 mrad operated at 300 kV, and the collection angle for the ADF detector was 50 to 200 mrad. The field of view is 5.5 nm with an image size of 512 × 512 pixels (10.4 pm/pixel). The beam current and the acquisition time were 54.8 pA and 83 ns/pixel, respectively, and the electron dose was 2624 e^−^ Å^−2^. We time-sequentially acquired 672 frames in 26.9 s, and the temporal resolution was 40 ms (*12*).

### Identification of Pt atom locations

The spatial drifts between frames (672 frames) were aligned by a cross-correlation algorithm (*21*). The present acquisition time per frame is 40 ms, which is much shorter than a standard acquisition time, and we, therefore, ignore a nonlinear spatial drift effect related to the microscope instability. However, it requires precise correction of the scan distortion (orthogonality) originating from the scan coil imperfection, which will be critical to the 3D reconstruction of the Pt trimer. Because we know the lattice parameters of the rutile TiO_2_, we corrected the slight scan distortion by an affine transformation, where all the frames were transformed by a single affine matrix determined from the time-averaged image of Fig. 1A. The quantum noise was suppressed by a Gaussian filter in reciprocal space. To explore the Pt atom locations, we first constructed a maximum intensity map, as shown in Fig. 1B. We have a 3D dataset of *I*(*x*, *y*, *t*), where the pixel position is (*x*, *y*) and the frame number is *t*. To construct at maximum intensity map, we selected a maximum value for each pixel in the whole frames: *I*_max_(*x*, *y*) = Max[*I*(*x*, *y*, *t*)]. When a Pt atom is located at a certain position even in a single frame, the pixel value should be the highest in the whole frames, and we can project the locations of Pt atoms in the maximum intensity map. The intensity contribution of the TiO_2_ substrate was removed by subtracting the time-averaged image of Fig. 1A from the respective frames, and the results are given in Fig. 1 (D to N).

After the subtraction of the time-averaged image from each frame, there appear several bright contrasts related to the noise, which may be Pt atoms. To safely identify the location of Pt atoms, we double-checked the original and neighboring frames. The 2D projected Pt atomic locations were determined by 2D Gaussian fitting with subpixel precision, which was used for the 3D reconstruction of the Pt trimer.

### Evaluation of surface diffusion coefficient

In the present dataset, there are many single atoms, but most of them have temporarily interacted with the other Pt single atoms, which is unsuitable for the mobility evaluation as an isolated Pt single atom. Moreover, the movement of single Pt atoms is relatively large, and it could be possible to exchange with the other Pt atoms. To rigidly track a single Pt atom, we selected a relatively stable (small mobility) and an isolated Pt single atom, and we tracked the movement of the single Pt atom for 3.08 s (77 frames: #279 to #355). To evaluate the mobility of the single Pt atom and the Pt trimer (Fig. 1), we use integrated displacement (*L*) with a projected Pt location at a frame *i* (*X_i_*)

$$L=\Sigma_{i}\sqrt{{\left(X_{i+1}-X_{i}\right)}^{2}}$$ (2)

where *X_i_* for the Pt trimer is the CoM of the Pt trimer. Figure S1A shows the integrated displacement (in angstrom) as a function of relative time (in seconds) for the single Pt atom and the Pt trimer, respectively. Within the time interval of 3.08 s, the integrated displacement for the single Pt atom and the Pt trimer is 13.5 and 45.7 Å, respectively. The velocity (or mobility) for the single Pt and the Pt trimer is estimated to be 4.38 and 14.9 Å s^−1^, respectively. The velocity of the single Pt atom is 3.4 times larger than that of the Pt trimer. We selected a relatively stable (less mobile) single Pt atom, suggesting that the mobility of a single Pt atom should be much larger than that of a Pt trimer. The observed Pt atom surface diffusion (2D) is random walk, and hence, the mean squared displacement is related to the diffusion coefficient (*D*)

$$4\mathrm{Dt}=\frac{1}{N}\Sigma_{i}{\left|X_{i+1}-X_{i}\right|}^{2}$$ (3)

In general, *N* must be the number of traced single Pt atoms (ensemble). However, we traced only one single Pt atom, and we here use time average (*N* = 77 frames) rather than ensemble average (ergodic theorem). With this assumption, the surface diffusion coefficient at 573 K for a single Pt atom and a Pt trimer is estimated to be 7.5 × 10^−19^ and 1.9 × 10^−22^ m^2^ s^−1^, respectively.

### Theoretical calculations

The calculations were performed using the projector augmented wave method implemented in the Vienna ab initio simulation package code (*22*, *23*). The exchange-correlation energy was approximated by the spin-polarized generalized-gradient approximation. The plane-wave cutoff energy was set to 500 eV. A (110) surface slab model was used to evaluate the adsorption energy. The slab thickness and vacuum thickness were set to 19.3 and 22.6 Å, respectively. To remove the spurious interaction between periodic images of Pt trimers, the slab was multiplied by two and three along the [1-10] and [001] directions, respectively. The Brillouin-zone sampling was performed using a 2 × 2 × 1 Monkhorst-Pack mesh. The adsorption energy of the Pt trimer (absorption energy per atom) on the TiO_2_ (110) surface was evaluated by the following equation

$$E_{\text{ad}}^{\text{coh}}=\left(E_{\text{TiO}2}+3E_{\text{Pt}}-E_{3\text{Pt}/\text{TiO}2}\right)/3$$ (4)

where *E*_TiO2_, *E*_Pt_, and *E*_3Pt/TiO2_ are the total energies of the pristine TiO_2_ surface slab, an isolated single Pt atom, and Pt trimer adsorbed on the TiO_2_ (110) surface, respectively.

### 3D structure retrieval of the Pt trimer on the TiO_2_ (110)

As confirmed by DFT calculations, it is reasonable to assume that the Pt trimer on the TiO_2_ (110) is an equilateral triangle. Under the constraint, the 3D atomic structure of the Pt trimer can be described by the four parameters: interatomic distance and Euler angles. We set the initial coordination of the reference equilateral triangle ABC as $A(0,\sqrt{3}a/3,0)$, $B(-a/2,\sqrt{3}a/6,0)$, and $C(a/2,-\sqrt{3}a/6,0)$, respectively, where the CoM is set to the origin. The 3D rotation matrix may be given by the Euler angles

$$\begin{array}{c} R\left(\theta ,\phi ,\psi \right)=\\ \left(\begin{array}{ccc} \text{cos}\;\phi \;\text{cos}\;\psi & \text{sin}\;\theta \;\text{sin}\;\phi \;\text{cos}\;\psi -\text{cos}\;\theta \;\text{sin}\;\psi & \text{cos}\;\theta \;\text{sin}\;\phi \;\text{cos}\;\psi +\text{sin}\;\theta \;\text{sin}\;\psi \\ \text{cos}\;\phi \;\text{sin}\;\psi & \text{sin}\;\theta \;\text{sin}\;\phi \;\text{sin}\;\psi +\text{cos}\;\theta \;\text{cos}\;\psi & \text{cos}\;\theta \;\text{sin}\;\phi \;\text{sin}\;\psi -\text{sin}\;\theta \;\text{cos}\;\psi \\ -\text{sin}\;\phi & \text{sin}\;\theta \;\text{cos}\;\phi & \text{cos}\;\theta \;\text{cos}\;\phi \end{array}\right) \end{array}$$ (5)

After the 3D rotation and scaling of the reference triangle, the projected positions of *A*(*x_A_*, *y_A_*) and *B*(*x_B_*, *y_B_*) are calculated as follows

$$\begin{array}{c} x_{A}=\frac{\sqrt{3a}}{3}\left(\text{sin}\;\theta \;\text{sin}\;\phi \;\text{cos}\;\psi -\text{cos}\;\theta \;\text{sin}\;\psi \right),\\ y_{A}=\frac{\sqrt{3a}}{3}\left(\text{sin}\;\theta \;\text{sin}\;\phi \;\text{sin}\;\psi +\text{cos}\;\theta \;\text{cos}\;\psi \right),\\ x_{B}=-\frac{a}{2}\text{cos}\;\phi \;\text{cos}\;\psi -\frac{\sqrt{3a}}{6}\left(\text{sin}\;\theta \;\text{sin}\;\phi \;\text{cos}\;\psi -\text{cos}\;\theta \;\text{sin}\;\psi \right),\\ y_{B}=\frac{a}{2}\text{cos}\;\phi \;\text{sin}\;\psi -\frac{\sqrt{3a}}{6}\left(\text{sin}\;\theta \;\text{sin}\;\phi \;\text{sin}\;\psi +\text{cos}\;\theta \;\text{cos}\;\psi \right) \end{array}$$ (6)

Because the CoM of the reference triangle is set to the origin, *C* is linearly dependent on *A* and *B* (*A* + *B* + *C* = 0), and we will not use the relationship on *C*. The experimental 2D projected positions of a triangle are denoted as ($x_{A}^{\mathrm{ex}}$, $y_{A}^{\mathrm{ex}}$), ($x_{B}^{\mathrm{ex}}$, $y_{B}^{\mathrm{ex}}$), and ($x_{C}^{\mathrm{ex}}$, $y_{C}^{\mathrm{ex}}$), respectively. We then obtain the following four nonlinear equations as $x_{A}^{\mathrm{ex}}$ = $x_{A}$, $y_{A}^{\mathrm{ex}}$ = $y_{A}$, $x_{B}^{\mathrm{ex}}$ = $x_{B}$, and $y_{B}^{\mathrm{ex}}$ = *y_B_*. To solve these nonlinear equations, we implement a nonlinear optimization algorithm (Broyden-Fletcher-Goldfarb-Shanno algorithm) of Eq. 1. Our algorithm has been tested for randomly generated many equilateral triangles, and we confirmed that the retrieved lattice parameters and Euler angles are precisely matched with the input ones.

## Supplementary Materials

### This PDF file includes:

Supplementary Text Figs. S1 and S2 Legends for movies S1 and S2 References
