# Supplementary material for: Real-time tracking of three-dimensional atomic dynamics of Pt trimer on TiO2 (110)
Source: Sci Adv. 2024 Feb 28;10(9):eadk6501. doi: 10.1126/sciadv.adk6501 (PMC10901364; doi:10.1126/sciadv.adk6501)
Supplement: Supplementary file 1 — Supplementary Text Figs. S1 and S2 Legends for movies S1 and S2 References [file sciadv.adk6501_sm.pdf]

Science Advances
AAAS

Supplementary Materials for

Real-time tracking of three-dimensional atomic dynamics of Pt trimer on TiO_2 (110)

Ryo Ishikawa *et al.*

Corresponding author: Ryo Ishikawa, ishikawa@sigma.t.u-tokyo.ac.jp

Sci. Adv. **10**, eadk6501 (2024)
DOI: 10.1126/sciadv.adk6501

The PDF file includes:

Supplementary Text
Figs. S1 and S2
Legends for movies S1 and S2
References

Other Supplementary Material for this manuscript includes the following:

Movies S1 and S2

Supplementary Text

Comparison with image simulation

Fig. S1A is ADF STEM of frame #77, which contains a Pt-trimer (white rectangle) and a single Pt atom (white arrowhead) emitted from the Pt cluster at the bottom left. The Z-contrast intensity of the trimer is similar to that of a single Pt atom, which can also be confirmed by the many other single Pt atoms in Supplemental Movie 1. To semi-quantitatively evaluate the Z-contrast intensity of Pt atoms, we performed image simulation using a multislice algorithm with the frozen phonon model (TEMSIM package) (24), where the optical condition of the microscope is the same as the experiment and the sample thickness is 10 nm. Fig. S1B and C show the high-magnified experimental image obtained from the white rectangle region in A and the simulated image containing a Pt trimer, respectively. Compared with the image simulation, the Z-contrast intensity of a Pt single atom is relatively lower than that in the simulated image, which is found in the intensity profiles along the X–Y direction in Fig. S1D. The Pt trimer is dynamically moving on the surface, and therefore, the Z-contrast intensity could be reduced. Moreover, the electron dose was considerably low, and the experimental image should have suffered from quantum noises, which makes it difficult to identify Pt single atoms. From these considerations, we conclude that the observed Pt cluster is a Pt trimer, and the Pt trimer is well isolated from the other single Pt atoms.

Electron beam irradiation effect

According to the literature, it is known that the sample heating effect via electron beam irradiation is negligible (25), especially at heating conditions. However, we need to consider the knock-on effect to enhance the surface diffusion of Pt atoms because the maximum energy transfer for a Pt atom at 300 kV is 4.0 eV. Here, we estimate the probability of energy transfer from an incident electron to a Pt atom via elastic collision using the Mott scattering cross section ($d\sigma_M(\theta)/d\Omega$) (9). If we know the activation energy for the Pt atom surface diffusion, the total cross-section can be calculated by integrating from the minimum scattering angle (θ_{min}) to π:

$$\sigma_{tot} = 2\pi \int_{\theta_{min}}^{\pi} \frac{d\sigma_M(\theta)}{d\Omega} sin\left(\frac{\theta}{2}\right). \quad \text{(S1)}$$

Fig. S1B shows the total cross-section of a Pt atom as a function of activation energy. The surface diffusion event rate (κ) induced by the electron beam irradiation is approximately given by the product of σ_{tot} and the number of incident electrons to a Pt atom (D_e) (Pt atom size ~ 1 Å^2):

$$\kappa = \sigma_{tot} \cdot D_e. \quad \text{(S2)}$$

In this experiment, we observed surface diffusion at relatively low dose conditions (D_e = 2624 e^- /Å^2). Although the Pt atom is a heavy element, the activation energy for the surface diffusion may be less than 1 eV. If the activation energy for the surface diffusion is 0.7 eV or 1 eV, then the surface diffusion assisted by electron beam irradiation will be every 2.9 and 7.5 frames, respectively. Therefore, the surface diffusion may be increased by 15 ± 5%. The observed surface diffusion should be mainly governed by specimen heating.

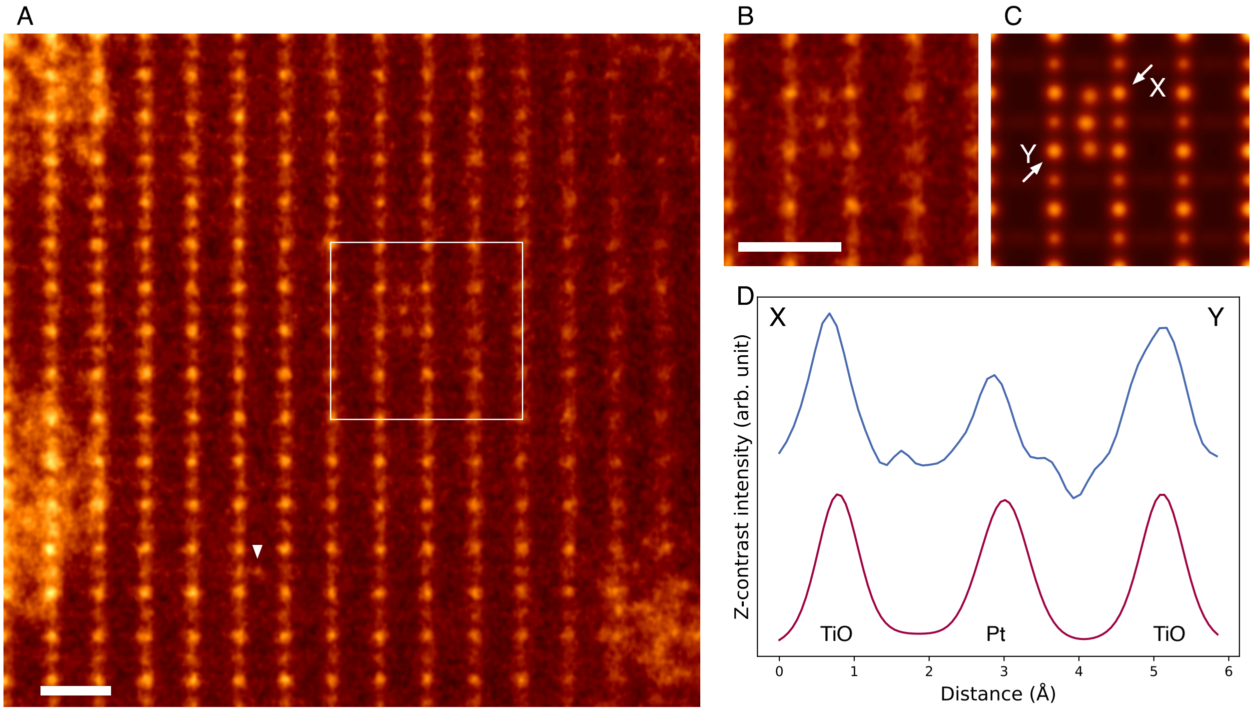

Fig. S1. Comparison between experimental and simulated images. (**A**) ADF STEM of frame #77 and (**B**) high-magnified image of the white rectangle is in (A). (**C**) Simulated ADF STEM image, including a Pt trimer on the TiO_2 (110) surface. (**D**) Z-contrast intensity profiles along the X-Y direction in (C) obtained from the experiment (upper) and the simulation (lower), respectively. The scale bars in A and B are 3 Å, respectively.

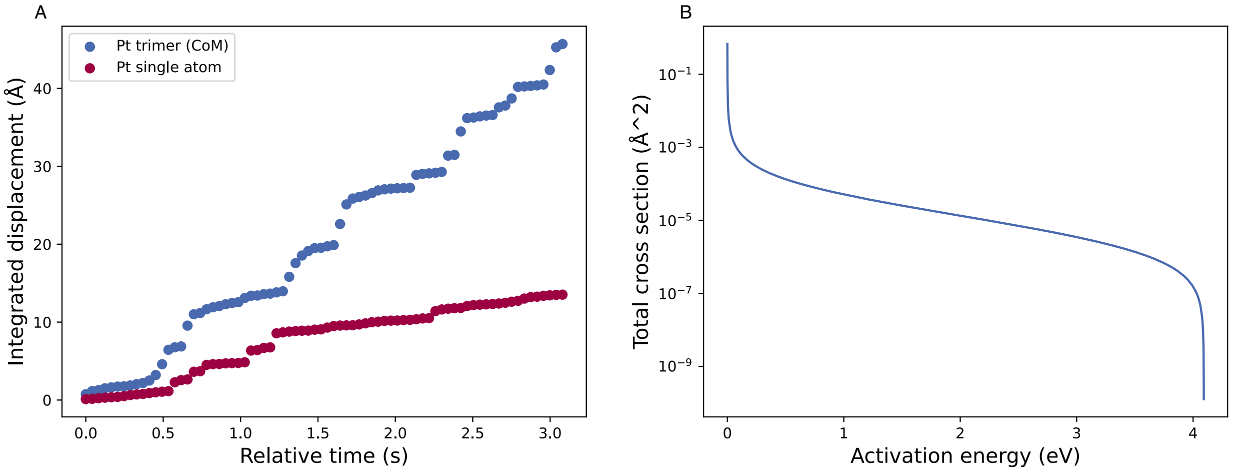

Fig. S2. A single Pt atom mobility and electron beam irradiation effect. (**A**) The integrated displacement of Pt trimer (center of mass) and a single Pt atom as a function of time. (**B**) Total cross-section of a single Pt atom as a function of activation energy.

Movie S1. High-spatiotemporal-resolution ADF STEM images of a Pt trimer on TiO_2 (110) surface. The movie contains the first 150 frames with a frame rate of 25 fps.

Movie S2. Time-average subtracted high-spatiotemporal-resolution ADF STEM images of a Pt trimer on TiO_2 (110) surface. To remove the TiO_2 lattice contrast, the time-averaged image was subtracted from the Suppl. Movie S1.

REFERENCES AND NOTES

1. B. Qiao, A. Wang, X. Yang, L. F. Allard, Z. Jiang, Y. Cui, J. Liu, J. Li, T. Zhang, Single-atom catalysis of CO oxidation using Pt_1/FeO_x. *Nat. Chem.* **3**, 634–641 (2011).

2. X.-F. Yang, A. Wang, B. Qiao, J. Li, J. Liu, T. Zhang, Single-atom catalysts: A new frontier in heterogeneous catalysis. *Acc. Chem. Res.* **46**, 1740–1748 (2013).

3. Y. Guo, M. Wang, Q. Zhu, D. Xiao, D. Ma, Ensemble effect for single-atom, small cluster and nanoparticle catalysts. *Nat. Catal.* **5**, 766–776 (2022).

4. X. Li, X. I. Pereira-Hernandez, Y. Chen, J. Xu, J. Zhao, C. W. Pao, C. Y. Fang, J. Zeng, Y. Wang, B. C. Gates, J. Liu, Functional CeO_x nanoglues for robust atomically dispersed catalysts. *Nature* **611**, 284–288 (2022).

5. C. Vogt, B. M. Weckhuysen, The concept of active site in heterogeneous catalysis. *Nat. Rev. Chem.* **6**, 89–111 (2022).

6. S. J. Pennycook, L. A. Boatner, Chemically sensitive structure-imaging with a scanning transmission electron microscope. *Nature* **336**, 565–567 (1988).

7. S. J. Pennycook, P. D. Nellist, *Scanning Transmission Electron Microscopy Imaging and Analysis* (Springer, 2011).

8. J. Lee, W. Zhou, S. J. Pennycook, J.-C. Idrobo, S. T. Pantelides, Direct visualization of reversible dynamics in a Si_6 cluster embedded in a graphene pore. *Nat. Commun.* **4**, 1650 (2013).

9. R. Ishikawa, R. Mishra, A. R. Lupini, S. D. Findlay, T. Taniguchi, S. T. Pantelides, S. J. Pennycook, Direct observation of dopant atom diffusion in a bulk semiconductor crystal enhanced by a large size mismatch. *Phys. Rev. Lett.* **113**, 155501 (2014).

10. Y. Yang, C.-C. Chen, M. C. Scott, C. Ophus, R. Xu, A. Pryor, L. Wu, F. Sun, W. Theis, J. Zhou, M. Eisenbach, P. R. C. Kent, R. F. Sabirianov, H. Zeng, P. Ercius, J. Miao, Deciphering chemical order/disorder and material properties at the single-atom level. *Nature* **542**, 75–79 (2017).

11. R. Ishikawa, N. Shibata, T. Taniguchi, Y. Ikuhara, Three-dimensional imaging of a single dopant in a crystal. *Phys. Rev. Appl.* **13**, 034064 (2020).

12. R. Ishikawa, Y. Jimbo, M. Terao, M. Nishikawa, Y. Ueno, S. Morishita, M. Mukai, N. Shibata, Y. Ikuhara, High spatiotemporal-resolution imaging in the scanning transmission electron microscope. *Microscopy* **69**, 240–247 (2020).

13. T.-Y. Chang, Y. Tanaka, R. Ishikawa, K. Toyoura, K. Matsunaga, Y. Ikuhara, N. Shibata, Direct imaging of Pt single atoms adsorbed on TiO_2 (110) surfaces. *Nano Lett.* **14**, 134–138 (2014).

14. R. Ishikawa, Y. Ueno, Y. Ikuhara, N. Shibata, Direct observation of atomistic reaction process between Pt nanoparticles and TiO_2 (110). *Nano Lett.* **22**, 4161–4167 (2022).

15. X.-Q. Gong, A. Selloni, O. Dulub, P. Jacobson, U. Diebold, Small Au and Pt clusters at the anatase TiO_2(101) surface: Behavior at terraces, steps, and surface oxygen vacancies. *J. Am. Chem. Soc.* **130**, 370–381 (2008).

16. N. Shibata, A. Goto, S.-Y. Choi, T. Mizoguchi, S. D. Findlay, T. Yamamoto, Y. Ikuhara, Direct imaging of reconstructed atoms on TiO_2 (110) surfaces. *Science* **322**, 570–573 (2008).

17. J. W. Arblaster, Crystallographic properties of platinum. *Platinum Metals Rev.* **41**, 12 (1997).

18. L. Pauling, *The Nature of the Chemical Bond* (Cornell Univ. Press, 1960).

19. S. Siegel, H. R. Hoekstra, B. S. Tani, The crystal structure of β-platinum dioxide. *J. Inorg. Nucl. Chem.* **31**, 3803–3807 (1969).

20. S. Morishita, R. Ishikawa, Y. Kohno, H. Sawada, N. Shibata, Y. Ikuhara, Attainment of 40.5 pm spatial resolution using 300 kV scanning transmission electron microscope equipped with fifth-order aberration corrector. *Microscopy* **67**, 46–50 (2018).

21. R. Ishikawa, A. R. Lupini, S. D. Findlay, S. J. Pennycook, Quantitative annular dark field electron microscopy using single electron signals. *Microsc. Microanal.* **20**, 99–110 (2014).

22. P. E. Blöchl, Projector augmented-wave method. *Phys. Rev. B* **50**, 17953–17979 (1994).

23. G. Kresse, D. Joubert, From ultrasoft pseudopotentials to the projector augmented-wave method. *Phys. Rev. B* **59**, 1758–1775 (1999).

24. E. J. Kirkland, *Advanced Computing in Electron Microscopy* (Springer, 2010).

25. L. Reimer, H. Kohl, *Transmission Electron Microscopy* (Springer, 2008).
